# Approaching Defect-free Amorphous Silicon Nitride by Plasma-assisted Atomic Beam Deposition for High Performance Gate Dielectric

**DOI:** 10.1038/srep28326

**Published:** 2016-06-21

**Authors:** Shu-Ju Tsai, Chiang-Lun Wang, Hung-Chun Lee, Chun-Yeh Lin, Jhih-Wei Chen, Hong-Wei Shiu, Lo-Yueh Chang, Han-Ting Hsueh, Hung-Ying Chen, Jyun-Yu Tsai, Ying-Hsin Lu, Ting-Chang Chang, Li-Wei Tu, Hsisheng Teng, Yi-Chun Chen, Chia-Hao Chen, Chung-Lin Wu

**Affiliations:** 1 Center for Micro/Nano Science and Technology, National Cheng Kung University, Tainan 70101, Taiwan; 2 Department of Physics, National Cheng Kung University, Tainan 70101, Taiwan; 3 National Synchrotron Radiation Research Center, Hsinchu 30076, Taiwan; 4 National Nano Devices Laboratories, National Applied Research Laboratories, Tainan 741, Taiwan; 5 Department of Physics, National Tsing-Hua University, Hsinchu 30013, Taiwan; 6 Department of Physics, National Sun Yat-Sen University, Kaohsiung 804, Taiwan; 7 Department of Chemical Engineering, National Cheng Kung University, Tainan 70101, Taiwan

## Abstract

In the past few decades, gate insulators with a high dielectric constant (high-k dielectric) enabling a physically thick but dielectrically thin insulating layer, have been used to replace traditional SiO_x_ insulator and to ensure continuous downscaling of Si-based transistor technology. However, due to the non-silicon derivative natures of the high-k metal oxides, transport properties in these dielectrics are still limited by various structural defects on the hetero-interfaces and inside the dielectrics. Here, we show that another insulating silicon compound, amorphous silicon nitride (*a*-Si_3_N_4_), is a promising candidate of effective electrical insulator for use as a high-k dielectric. We have examined *a*-Si_3_N_4_ deposited using the plasma-assisted atomic beam deposition (PA-ABD) technique in an ultra-high vacuum (UHV) environment and demonstrated the absence of defect-related luminescence; it was also found that the electronic structure across the *a*-Si_3_N_4_/Si heterojunction approaches the intrinsic limit, which exhibits large band gap energy and valence band offset. We demonstrate that charge transport properties in the metal/*a*-Si_3_N_4_/Si (MNS) structures approach defect-free limits with a large breakdown field and a low leakage current. Using PA-ABD, our results suggest a general strategy to markedly improve the performance of gate dielectric using a nearly defect-free insulator.

The challenge of finding a high quality gate dielectric on silicon and prospective 2D materials that exhibits ultrathin equivalent oxide thickness (EOT) and low interface defect density has been a fundamental problem that must be solved in order to continue to meet Moore’s law in the present semiconductor and post-silicon electronics. Insulators with high dielectric constants allow a high capacitance with a thicker film that reduces the direct tunneling leakage current. Recently, a wide variety of metals oxides (for example, HfO_2_, TiO_2_, Al_2_O_3_, La_2_O_3_, Ta_2_O_5,_ and SrTiO_3_) have been considered as possible replacements of SiO_2_^1^,^2^,^3^,^4^,^5^. However, these non-Si-based dielectrics generally exhibit poor interfaces with silicon that can markedly degrade the gate dielectric performance, thus need to consist a very thin SiO_2_ to reduce the interfacial defect density near the substrate^6^. Therefore, the use of an amorphous- phased silicon-based derivative is more realistic for the complete replacement of amorphous SiO_2_ in the gate dielectric. Amorphous silicon nitride (*a*-Si_3_N_4_) is one such material with a dielectric constant that is approximately twice the dielectric constant of SiO_2_, and has excellent mechanical, thermal, and electronic insulating properties^7^,^8^. Besides, *a*-Si_3_N_4_ has been demonstrated recently for an attractive alternative for 2D material field-effect transistors (FETs)^9^,^10^,^11^ because it shows less degradation on channel mobility compared to SiO_2_^12^ and HfO_2_^13^. Therefore, the improvement in EOT downscaling for Si_3_N_4_ is critical to be implemented for future generation of transistors. A thorough understanding of the conventional *a*-Si_3_N_4_ deposition process is required to find out the solution for lower EOT.

In traditional chemical vapor deposition (CVD) processes of *a*-Si_3_N_4_, the hydrogen/oxygen-radicals create an inherently large concentration of defects and thus lead to dielectric charging, which in turn governs either the operation or time-dependent performance degradation in gate dielectrics^14^,^15^,^16^. Therefore, a new option of deposition method should be available through an ultra-high vacuum (UHV) physical deposition process: in an extremely pure and hydrogen-free environment that contains only the constituent elements (Si, N) for *a*-Si_3_N_4_ deposition, and has extendibility to very large substrates with carefully source-substrate geometry design for both Si and N atoms having good uniformity of arrival rate over entire substrate area. Here, we demonstrate *a*-Si_3_N_4_ prepared via a one-step (without a post-annealing process), two atomic beams (Si evaporated beam and N_2_-plasma) method called plasma-assisted atomic beam deposition (PA-ABD) for use as a long expected and versatile insulating layer that meets a wide range of requirements for high-performance gate dielectrics. We show that the use of PA-ABD enables the formation of an atomically abrupt interface and a smooth surface *a*-Si_3_N_4_/Si heterojunction with excellent insulating properties such as a large dielectric band gap (~6 eV) and large heterojunction valence/conduction band offset (VBO/CBO) values for blocking mobile carriers while applying gate voltage. Consequently, under the current-voltage (I-V) characteristic measured on the metal/*a*-Si_3_N_4_/Si (MNS) heterostructure, the nearly defect-free nature inherent to the UHV PA-ABD process shows the scalability of the high-quality *a*-Si_3_N_4_ insulating layer with a large breakdown field and a low leakage current.

## Results

**Figure 1:**
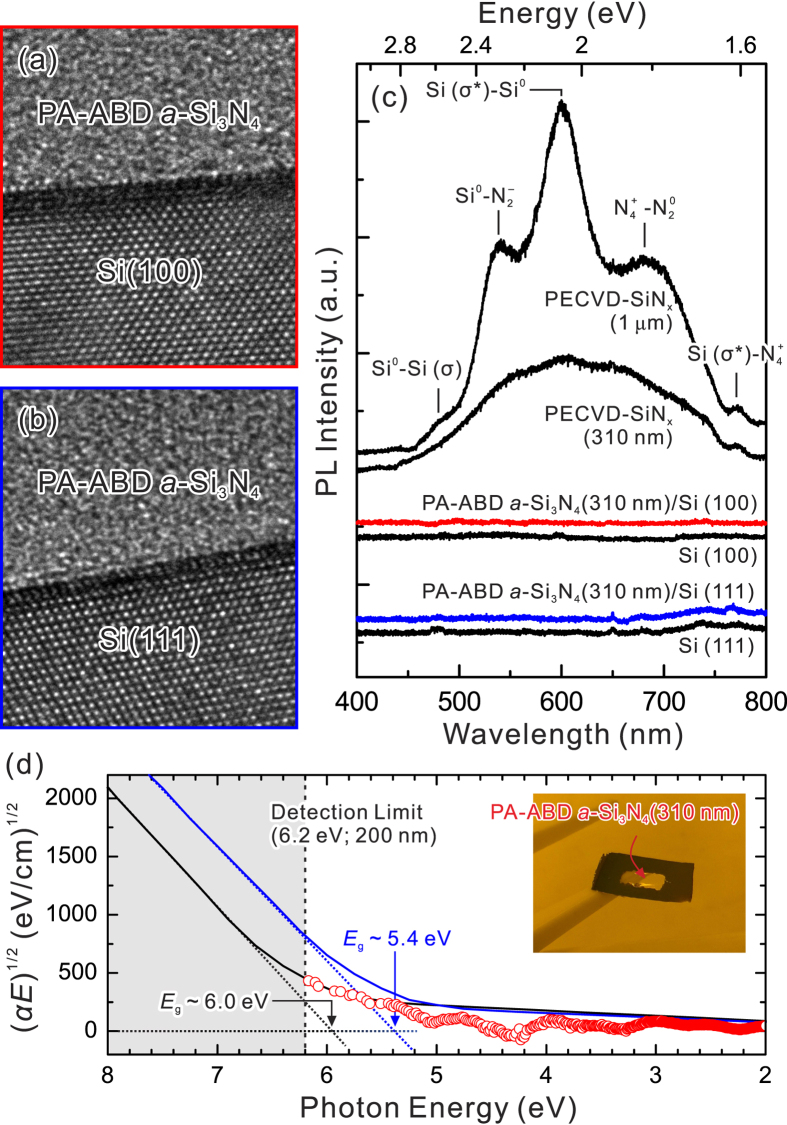
TEM, PL, and optical *E*_g_ characterizations: (**a**,**b**) are cross-sectional HRTEM images of PA-ABD *a*-Si_3_N_4_ grown on Si (100) and Si (111) substrates with pre-treatment at high temperature (600 °C) and lower temperature (950 °C), respectively. (**c**) Room temperature PL spectra for the PA-ABD *a*-Si_3_N_4_/Si (100) (red), the PA-ABD *a*-Si_3_N_4_/Si (111) (blue), the PECVD *a*-SiN_x_/Si (111) and bare Si (100) and Si (111) substrates (black curve). (**d**) Optical *E*_g_ measurement of PA-ABD *a*-Si_3_N_4_ with red circles for experimental data, black curve for fitting) by UV-visible absorption spectra. The blue curve is previously reported data for the stoichiometric *a*-Si_3_N_4_^23^. The inset is the photo of the PA-ABD *a*-Si_3_N_4_ membrane for the transmission measurement.

The cross-sectional high-resolution transmission electron microscopy (HR-TEM) images show that atomically abrupt amorphous/crystalline hetero-interfaces are formed on the reconstructed Si (111) surface (Fig. 1b) and the unreconstructed Si (100) surface (Fig. 1a), indicating higher electronic performance of the *a*-Si_3_N_4_/Si heterojunction resulting from the lower density of interface traps. We then focused on measuring the defects states inside the *a*-Si_3_N_4_ layer that directly determine the electronic properties; this was performed with room-temperature photoluminescence (PL) using a 325 nm excitation light source from a He–Cd laser. The PL spectra shown in Fig. 1c can characterize the electronic density of defect states and provide information about chemical bonding and the composition of defects. For comparison, *a*-SiN_x_ films deposited by conventional plasma-enhanced chemical vapor deposition (PECVD) with thicknesses of 1 μm and 310 nm are shown, exhibiting strong PL emission for wavelengths in the 450–800 nm range, that arises the electronic transition between gap in states of Si-Si and N-N bonds, and Si and N dangling bonds^17^,^18^,^19^. The 1-μm-thick *a*-SiN_x_ showed clear peaks at about 2.6 eV^17^, 2.3 eV^17^,^19^, 2.1 eV^17^, 1.8 eV^17^, and 1.6 eV^17^, that are associated with the defect optical transitions of Si^0^-Si(σ), Si^0^-N_2_^−^, Si(σ^*^) − Si^0^, N_4_^+^ − N_2_^0^, and Si(σ^*^) − N_4_^+^, respectively. In contrast to the strong defect emissions from the typical PECVD grown *a*-SiN_x_, the PL spectra from the PA-ABD grown *a*-Si_3_N_4_ on both (111) and (100) substrates are almost identical to the PL spectra of their substrates, demonstrating that the electrically active defects with atomic configurations that give rise to the electronic states in the *a*-Si_3_N_4_ band gap was substantial reduced by the absence of Si-H and N-H bonding during deposition^20^.

Furthermore, due to the presence of the gap states, *a*-SiN_x_ films grown by PECVD methods exhibit varying optical band gaps from about 2.4 eV to 5.4 eV, depending on the film composition process^17^,^21^,^22^. Thus, naturally, it is interesting to know whether the optical band gap of PA-ABD *a*-Si_3_N_4_ can be further increased if the defects that generate the gap states are almost entirely absent. Two optical measurements, transmission for the PA-ABD *a*-Si_3_N_4_ membrane and reflection for both PA-ABD *a*-Si_3_N_4_ and PECVD *a*-SiN_x_ (shown in Supplementary Fig. S1), were used to determine the optical band gaps. The PA-ABD *a*-Si_3_N_4_ membrane was fabricated by etching away the 2 × 4 mm^2^ area of the Si (100) substrate using a 49% KOH solution at 120 °C, as shown in the inset of Fig. 1d, followed by the measurement of the transmission (T) with a UV-Visible spectrophotometer. The optical band gap can be determined by plotting the absorption coefficient (α) against the photon energy (eV) and taking the intercept of the extrapolation to zero absorption with the photon energy axis (dashed lines in Fig. 1d). The detection limit of the spectrophotometer is 6.2 eV of photon energy, where the dramatic increase of absorption necessary for obtaining the intercept does not appear for PA-ABD *a*-Si_3_N_4_. Thus, absorption for the high photon energy region (>6.2 eV) is obtained by fitting the experimental PA-ABD *a*-Si_3_N_4_ data (red circles) with a blue shift of about 0.6 eV for the band gap (*E*_g_) of 5.4 eV of the well-known *a*-SiN_x_^23^. Reflectivity measurements were carried out to determine that the *E*_g_ for PECVD *a*-SiN_x_ is 3.1 eV for the comparison shown in Fig. 1d. Moreover, the *E*_g_ of PA-ABD *a*-Si_3_N_4_ is determined to be about 6.0 eV, which is consistent with the value obtained by both transmission and reflection measurements (see Supplementary Fig. S1); to the best of our knowledge, this value is closest to the theoretical *E*_g_ = 6.77 eV of the crystalline *β*-Si_3_N_4_ with no near band-edge states attributed to the N- or Si-dangling bonds extending from the CB or VB to reduce the *E*_g_^24^,^25^.

**Figure 2:**
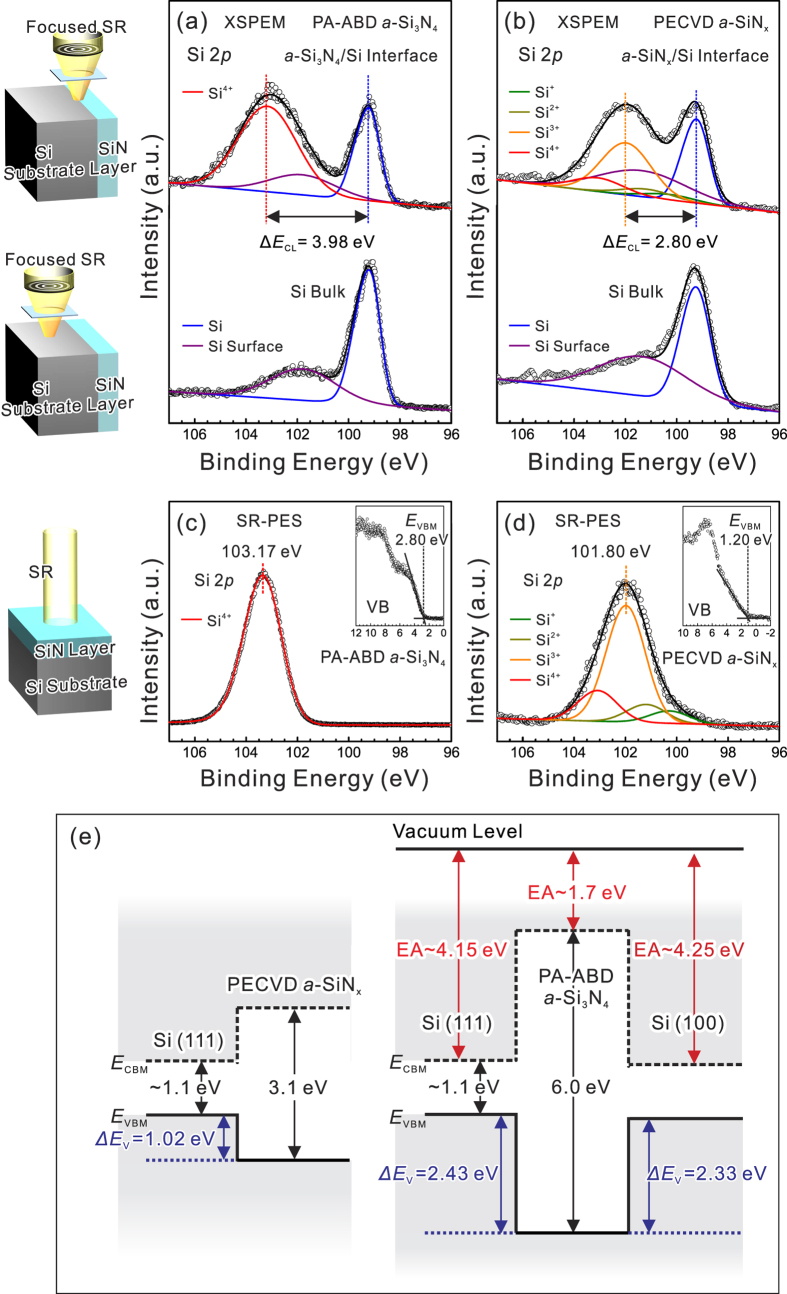
Band structure analysis: *μ*-PES spectra of the Si 2*p* core-level taken on cross-sectional PA-ABD *a*-Si_3_N_4_/Si(111) (**a**) and PECVD *a*-SiN_x_/Si(111) (**b**) samples for clean interface and bulk regions. SR-PES spectra of the Si 2*p* core-level taken on PA-ABD *a*-Si_3_N_4_ (**c**) and PECVD *a*-SiN_x_ (**d**) surfaces for obtaining energy differences between the Si 2*p* core-level and the valence-band maxima (*E*_CL_-*E*_VBM_). Schematic illustration of the XSPEM and SR-PES on the PA-ABD *a*-Si_3_N_4_/Si(111) and PECVD *a*-SiN_x_/Si(111) samples are included in the left panel of the Figure. The corresponding decomposition of Si 2*p* states for *a*-Si_3_N_4_ and *a*-SiN_x_ are also shown. The leading edges of valence bands for determining *E*_VBM_ from *a*-Si_3_N_4_ and *a*-SiN_x_ can be observed in the insets of (**c**,**d**), respectively. The black dots are the experimentally spectral data points and color lines are the curve-fitting results. (**e**) The schematic energy band diagram of *a*-SiN_x_/Si(111), *a*-Si_3_N_4_/Si(111), and *a*-Si_3_N_4_/Si(100) based on the measured values of Δ*E*_CL_, *E*_CL_-*E*_VBM_, in (**a**–**d**), and the crystalline orientated EA value of Si bulk.

Even if the nearly defect free structure is known, the connection between the atomically abrupt amorphous/crystalline interface at the *a*-Si_3_N_4_/Si heterojunction and its electronic structure is neither direct nor obvious. To directly visualize the electronic structure at the amorphous/crystalline hetero-interface, scanning photoelectron microscopy and spectroscopy (SPEM/S, National Synchrotron Radiation Research Center (NSRRC), Hsinchu, Taiwan) was used to provide the required spatial resolution (~sub-μm) for chemical mapping and the energy resolution (~50 meV) for localized photoelectron spectroscopy (*μ*-PES) on the cross-sectional *a*-Si_3_N_4_/Si(111) sample, here called cross-sectional SPEM (XSPEM). In particular, the Si (111) substrate enables *in situ* cleavage under an UHV environment for obtaining clean (no SiO_x_ layer) and smooth exposure surface (i.e., the (100) plane), as schematically shown in the left panel of Fig. 2. The *μ*-PES spectra taken from the hetero-interface region at different cross-sectional surfaces of the PA-ABD *a*-Si_3_N_4_/Si(111) and the PECVD *a*-SiN_x_/Si(111) heterojunctions are shown in Fig. 2a,b, respectively. The differences in Si 2*p* core-levels between *a*-Si_3_N_4_, *a*-SiN_x_, and the Si substrate can be analyzed by curve fitting using Voigt functions. For stoichiometric *a*-Si_3_N_4_ grown by PA-ABD, we find that the main chemical component of silicon nitride is the stoichiometric Si^4+^ valence state without the subnitride state, and one extra surface component in the low binding energy side is added to bulk silicon to obtain reasonably good fits^26^. Compared to PA-ABD *a*-Si_3_N_4_, one of the main differences in PECVD *a*-SiN_x_ is that it involves various subnitride components in the Si 2*p* spectrum and reveals its non-stoichiometric nature. The Si^3+^ component dominates in the Si 2*p* core-level spectrum because the hydrogen-containing nitridation process of PECVD results in incomplete nitridation and structural defects, leading to a binding energy shift of 2.80 eV relative to the energy reference of bulk Si^27^,^28^. The remaining subnitride components of Si^4+^, Si^2+^, and Si^+^ show shifts of 3.80, 1.90, and 1.00 eV, respectively^29^,^30^. In summary, according to the best spectral fit, the deconvoluted Si 2p core-level spectra clearly indicate a huge deviation in the binding energy difference between Si 2*p* bulk and stoichiometric *a*-Si_3_N_4_ and non-stoichiometric *a*-SiN_x_ core-level emissions of about 1.2 eV (∆*E*_CL_(*a*-Si_3_N_4_) = 3.98 eV and ∆*E*_CL_(*a*-SiN_x_) = 2.80 eV).

To determine the valence band offset (VBO) values of different silicon-nitride/Si heterojunctions, it is necessary to measure the binding energy differences (*E*_CL_ − *E*_VBM_) of the dominant Si core-level with respect to the corresponding valence band maximum (VBM) positions in *a*-Si_3_N_4_ and *a*-SiN_x_ films. The measured *E*_CL_ − *E*_VBM_ value of PA-ABD *a*-Si_3_N_4_ is about 100.37 eV (Fig. 2c), and that for PECVD *a*-SiN_x_ is about 100.60 eV (Fig. 2d), both samples were contamination and native-oxide free following chemical cleaning by diluted HF solutions and then rinsing with deionized water. In combination with the measured *E*_CL_ − *E*_VBM_ value of the Si substrate of about 98.82 eV (shown in Supplementary Fig. S2), which is a material constant, the VBO values can be determined using the relation ∆*E*_V_ = (*E*_CL_ − *E*_VBM_)Si − (*E*_CL_ − *E*_VBM_)SiN + (∆*E*_CL_)SiN/Si, resulting in type-I VBOs of 2.43 eV and 1.02 eV for the PA-ABD *a*-Si_3_N_4_/Si (111) and the PECVD *a*-SiN_x_/Si (111) heterojunction (Fig. 2e), respectively. Taking the band gap values of *a*-Si_3_N_4_ and *a*-SiN_x_ to be equal to 6.0 eV and 3.1 eV, respectively, the nearly symmetry in band alignment (VBO = 2.43 and CBO = 2.47) of the *a*-Si_3_N_4_/Si (111) heterojunction is shown in Fig. 2e, indicating the absence of defects exhibits large VBO/CBO values. Moreover, the VBO of the *a*-Si_3_N_4_/Si (100) heterojunction can be obtained by using the crystalline dependent electron affinity (EA) value of the bulk Si, which is ~4.15 eV for Si (111) and ~4.25 eV for Si (100)^31^,^32^. As illustrated in Fig. 2e, by aligning the vacuum levels of Si (111), Si(100), and *a*-Si_3_N_4_, the VBO of the *a*-Si_3_N_4_/Si(100) heterojunction can be determined to be 2.33 eV, implying an EA of about 1.7 eV for *a*-Si_3_N_4_, similar to the previously reported value of 1.8 ± 0.1 eV^33^.

**Figure 3:**
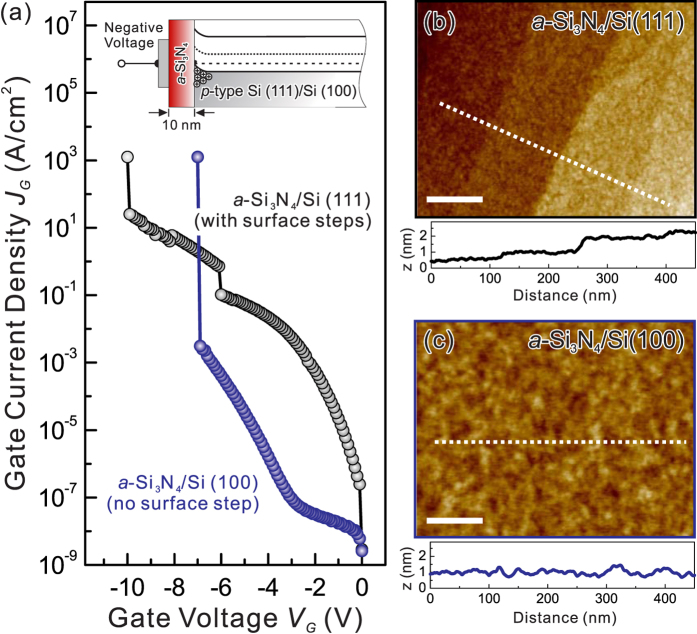
Leakage current and surface morphology characterizations: (**a**) Leakage gate current densities and breakdown fields for the 10-nm thick PA-ABD *a*-Si_3_N_4_ grown on Si (111) (black curve) and Si (100) (blue curve). The top inset is the schematic energy band diagram of the MNS capacitor with a *p*-type substrate for a negative gate bias. The AFM images taken on the corresponding *a*-Si_3_N_4_ layers with (**b**) and without (**c**) surface steps grown on Si (111) and Si (100), respectively. The average height profiles are shown below the images and the scale bars indicate for 100 nm of length.

The obtained PA-ABD *a*-Si_3_N_4_/Si heterojunction offers a nitride dielectric with insulating properties that have significant implications for application in electronic devices in that it can provide a sufficient energy barrier for both electron and hole injection. However, as shown in Fig. 3a, the leakage current characteristics of the metal/10-nm-thick *a*-Si_3_N_4_ (insulator)/Si(111) (MNS) structure having the desired high band offset and low interfacial defect states are highly unfavorable; namely, the leakage current is quite high for the *a*-Si_3_N_4_/Si(111) heterojunction with smooth surface morphology compared with atomic surface step (shown in Fig. 3b). Recently, large-scale molecular dynamics (MD) simulations have been performed to study the structure, dynamics, and mechanical behavior of the *a*-Si_3_N_4_/Si(111) heterostructure^34^,^35^. The *a*-Si_3_N_4_ exhibits stress nano-domains at the interface with Si (111), which would result in uneven dopant distribution that is then transferred into the *a*-Si_3_N_4_ layer with a thickness on the order of few nm. The observation of a large leakage current of MNS on a Si (111) substrate is in good support of the large-scale MD predictions that the interfacial stress pattern leads to the formation of a dopant pattern in the entire thin *a*-Si_3_N_4_ layer, which is indeed a problem for using *a*-Si_3_N_4_ as a good gate dielectric.

To avoid stress pattern formation and match the Si industry requirement of a Si (100) crystalline substrate, the MNS structure with 10-nm-thick *a*-Si_3_N_4_ was prepared on a Si (100) substrate by PA-ABD, showing a surface roughness (~0.13 nm, shown in Fig. 3c ) identical to that of *a*-Si_3_N_4_ grown on Si (111). As seen in Fig. 3a, the leakage characteristics of MNS on Si (100) substrate show great improvement with a leakage reduction of about 5 orders of magnitude compared to the MNS on Si (111) at −3 V. Moreover, this voltage ramp test indicates that the *J*_G_ of the MNS structure with 10-nm-thick *a*-Si_3_N_4_ can be maintained up to a breakdown field (*E*_BD_) as high as 7 MV/cm, before it begins to exhibit breakdown at about −7 V. In addition, the *E*_BD_ was 7~8.3 MV/cm for thicker layers of PA-ABD *a*-Si_3_N_4_ (10~50 nm, shown in Supplementary Fig. S3), and the lower breakdown field of the PA-ABD *a*-Si_3_N_4_ (*k* = 7.8) compared to that of 15 MV/cm for SiO_x_ (*k* = 3.9) is in good agreement with an approximate *E*_BD_ ~ *k*^−1/2^ dependence^36^.

**Figure 4:**
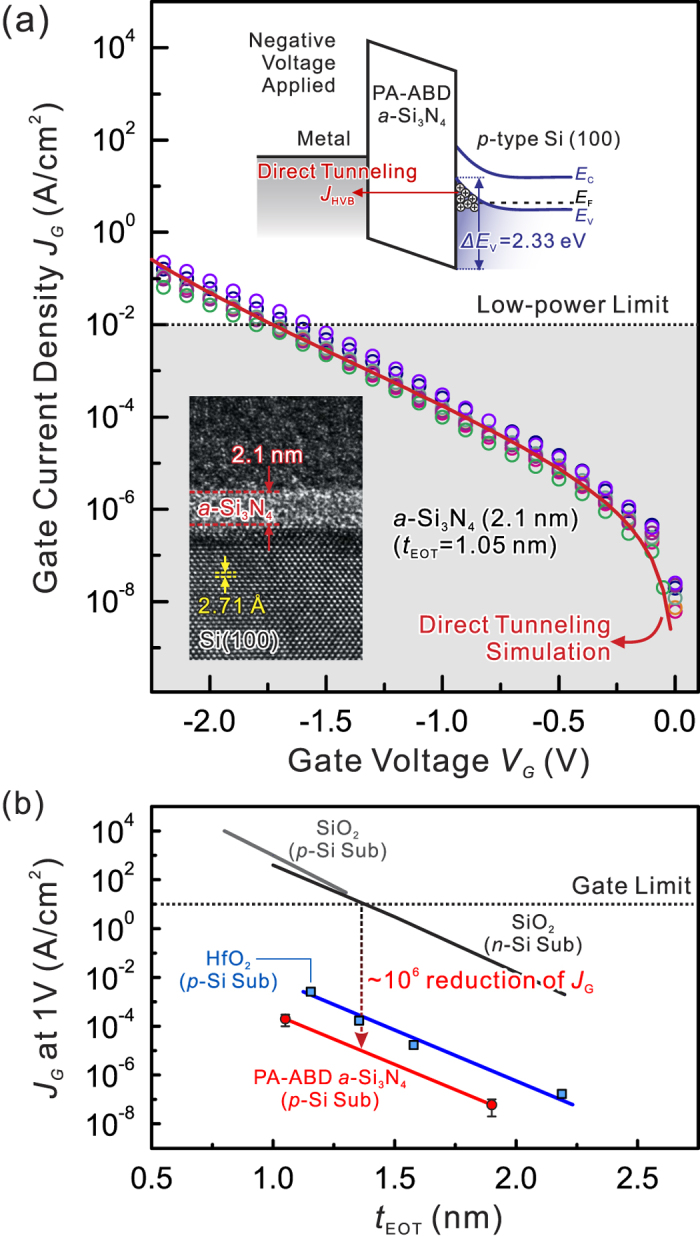
Gate current densities modeling and EOT analysis: (**a**) Model the gate current densities for 2.1 nm of PA-ABD *a*-Si_3_N_4_ on the *p*-type Si (100) substrate with dots for experimental data and with red curve for the model of direct tunneling. The top inset is the energy band diagram of the MNS capacitor with a *p*-type substrate for a negative gate bias, and the bottom inset is cross sectional HR TEM image. (**b**) Plot of leakage current density at 1V of gate voltage versus EOT for SiO_2_^41^, HfO_2_^42^,^43^, and PA-ABD *a*-Si_3_N_4_ for comparison, and PA-ABD *a*-Si_3_N_4_ shows a significant leakage current reduction. The criteria of low power limit and gate limit of leakage current densities are taken from previous reports^1^,^44^.

To further elucidate the insulating properties of the ultrathin PA-ABD *a*-Si_3_N_4_ layer, its charge transport mechanism was investigated by measuring *J*_G_ − *V*_G_ characteristics using the MNS device with 2.1 nm-thick *a*-Si_3_N_4_ layers (equivalent oxide thickness (EOT) = *t* × (*k*_SiOx_/*k*_*a*-Si3N4_) = 1.05 nm, where *t* is thickness and *k*_SiOx_ = 3.9). The EOT values shown in Fig. 4 were also confirmed by capacitance-voltage measurements on the MNS devices. The *J*_G_ − *V*_G_ characteristics of seven MNS devices (Fig. 4a) over a negative *V*_G_ range from 0 to −2.5 V are all well matched with the direct hole tunneling from valence band (HVB) model with the large VBO = 2.33 eV (shown in Fig. 2e for the *a*-Si_3_N_4_/Si(100) heterojunction) and *m*_h_^*^ = 0.77, indicating that charge transport through the *a*-Si_3_N_4_ layer is governed by tunneling and can be observed only when the defect density is sufficiently low so that thermally assisted conduction is suppressed significantly. This leads to a remarkable reduction of the *J*_G_ of the ultrathin *a*-Si_3_N_4_ by 4 orders of magnitude compared with *J*_G_ values reports for other *a*-SiN_x_ films and enables a wider *V*_G_ range for low-power operation^37^,^38^. Furthermore, no breakdown of PA-ABD *a*-Si_3_N_4_ for 2.1 nm is caused by the tunneling currents flowing though the dielectric due to direct tunneling, thus lowering the strength of the local electric field in the dielectric layer. Moreover, the observation of no breakdown for ultrathin PA-ABD *a*-Si_3_N_4_ is a signature of the relatively low trap/defect density in the dielectric and the superior smoothness and uniformity at the *a*-Si_3_N_4_/Si interface, that prevent the formation of the conduction path across the dielectric for breakdown^39^,^40^. Additionally, the gate leakage *J*_*G*_ of the MNS structure as a function of EOT for the *a*-Si_3_N_4_ layer thickness at 1 V is plotted in Fig. 4b with conventionally used dielectrics of SiO_2_ and HfO_2_ for comparison, clearly indicating that the *J*_*G*_ value of *a*-Si_3_N_4_ is a factor of 10^6^ and 10^1^~10^3^ lower than that of SiO_x_^41^ and HfO_2_^42^,^43^ at the same EOT, respectively. The extrapolation of leakage currents *J*_G_ suggests that *a*-Si_3_N_4_ with an EOT of 0.9 and 0.5 nm could replace SiO_x_ for 7 nm of physical gate length (18-nm node) of low standby power and high power devices, respectively^1^,^44^. These observations support of the hypothesis that the hydrogen-free nature of PA-ABD process leaves no N-H residues in the final *a*-Si_3_N_4_ films thus making them less prone to defect formation. Furthermore, the EOT downscaling to 0.5 nm and wafer-scale fabrication capabilities of PA-ABD *a*-Si_3_N_4_ will provide an ideal dielectric platform with defect-free nature for future 2D materials-based electronics owning to higher channel mobility compared to intrinsically defect-containing SiO_2_^9^,^10^,^11^ and other high-*k* metal oxides, such as HfO_2_^45^, ZrO_2_^46^, and Y_2_O_3_^47^.

## Discussion

In summary, to our knowledge, this work is the first demonstration of the defect-free *a*-Si_3_N_4_ insulating layer for use as a gate dielectric on Si with an extremely large band gap energy and a low leakage current. Based on the precise control of thickness, residual-free nature, reacted-element-only environment, and low thermal budget, the PA-ABD growth technique can meet the requirements such as ultrathin film thickness demanded by emerging nano-CMOS transistors, especially taking into account the good barrier nature of silicon nitride for boron dopant penetration. Moreover, PA-ABD *a*-Si_3_N_4_ is promising for complete replacement of SiO_2_ in the gate stack due to the same natural advantages offered to the industry by the extremely low interface/interlayer defect density.

## Methods

### Silicon nitride deposition

We grew *a*-Si_3_N_4_ first on Si (111) and then on Si (100) 2-inch wafer substrates; both substrates were *p*-type with a resistivity of 5–10 Ωcm. The wafers were cleaned by a standard RCA clean procedure, and dilute HF etching was then used to remove surface contaminations and the native oxide on the Si surface. The cleaned Si wafers were loaded into an ultra-high-vacuum (UHV) chamber (less than 1 × 10^−10 ^torr) equipped with a reflection high-energy electron diffraction (RHEED) system for *in-situ* determination of the surface atomic structure, a conventional thermally heated effusion cell for Si, and a radio-frequency (rf) plasma source for nitrogen. Remarkably, preparing the Si (111) surface by typical chemical wet etching and subsequent annealing at 950 °C in the UHV environment leads to the formation of the 7 × 7 surface reconstructed structure, and Si (100) surface leads to the formation of the 1 × 1 surface structure under subsequent annealing at 600 °C, where both determined surface structures agree with the lack of bonds to surface contaminants. Subsequently, the PA-ABD *a*-Si_3_N_4_ films were deposited upon both the (111) and (100) substrates at 550 °C with N atomic beam by using an rf-plasma source of pure nitrogen gas (2 × 10^−5 ^torr) with a power of 550 W, with Si atomic beam with flux at 5 × 10^−9 ^torr, and with a low growth rate of about 0.1 nm/min. The PECVD a-SiNx (Oxford Plasmalab System 100) is grown at 300 °C substrate temperature, at 25 W of rf power, at 800/25/8 sccm of N_2_/SiH_4_/NH_3_ flow, and at a pressure of 1 torr.

### Characterization of silicon nitride band gap

Photoluminescence (PL) spectra were performed with a spectrometer (LabRAM HR, Jobin Yvon), and transmission and reflection measurement were using a UV-Visible spectrophotometer (U-4100, Hitachi High-Tech. Corp.). The PA-ABD *a*-Si_3_N_4_/Si (111) with pretreatment at high temperature and PECVD *a*-SiN_x_ are not resist to KOH, and thus not able to perform the transmission experiment on these samples. The optical band gap was determined from the expression given by Tauc for amorphous materials:^48^


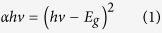


where *α* is the absorption coefficient given by *α* = 2.303 × log(T/d) (d is the thickness of PA-ABD *a*-Si_3_N_4_), hv is incident photon energy, and *E*_g_ the optical band gap.

Scanning photoelectron spectroscopy/microscopy characterization:

The SPEM/S system used here utilizes a combination of a Fresnel zone plate and an order-sorting aperture to focus the monochromatic (380 eV) soft x-ray with a beam size of about 100–200 nm. By setting the electron collecting energy window of the multiple-channel hemispherical electron energy analyzer while scanning the *a*-Si_3_N_4_/Si(111) heterojunction, a two-dimensional distribution of that particular core-level can be mapped. After acquiring the SPEM images, the focused beam was moved to specific locations to perform high-energy-resolution (~50 meV), microscopic-area photoelectron spectroscopy (μ-PES).

### Device fabrication

The MNS capacitors were fabricated by depositing 50 nm Ti/150 nm Au on silicon nitride as top gate electrode using a shadow mask with an area of ~5000 μm^2^. Then, the backside of p-Si substrate was polished to remove native oxide and 200 nm Pt was deposited as bottom electrode.

### Electrical characterization of MNS structure

Leakage current densities for PA-ABD a-SiNx were measured on metal-insulator-semiconductor (MNS) capacitors using a semiconductor parameter analyzer (B1500, Keysight Technology) and a probe station (M150, Cascade).

### Direct tunneling simulation for MNS structure

The direct tunneling gate leakage current (*J*_*HVB*_) can be described by^49^


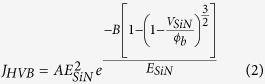


where 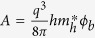, 
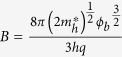
, *q* is electronic charge, *h* is Plank’s constant, *ϕ*_b_ (eV) is VBO between *a*-Si_3_N_4_ and Si substrate, 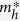 is hole effective mass for *a*-Si_3_N_4_, and *E*_SiN_ (=*V*/*d*, *V* for gate bias, and *d* for thickness of *a*-Si_3_N_4_) is electric field in the dielectric.

## Additional Information

**How to cite this article**: Tsai, S.-J. *et al*. Approaching Defect-free Amorphous Silicon Nitride by Plasma-assisted Atomic Beam Deposition for High Performance Gate Dielectric. *Sci. Rep.* **6**, 28326; doi: 10.1038/srep28326 (2016).

## Supplementary Material

Supplementary Information
