# Supplementary material for: Approaching Defect-free Amorphous Silicon Nitride by Plasma-assisted Atomic Beam Deposition for High Performance Gate Dielectric
Source: Sci Rep. 2016 Jun 21;6:28326. doi: 10.1038/srep28326 (PMC4915203; doi:10.1038/srep28326)
Supplement: Supplementary Information [file srep28326-s1.pdf]

Approaching Defect-free Amorphous Silicon Nitride by Plasma-assisted Atomic Beam Deposition for High Performance Gate Dielectric

Shu-Ju Tsai[1*], Chiang-Lun Wang[2], Hung-Chun Lee[2], Chun-Yeh Lin[2], Jhih-Wei Chen[2], Hong-Wei Shiu[3], Lo-Yueh Chang[3], Han-Ting Hsueh[4], Hung-Ying Chen[5], Jyun-Yu Tsai[6], Ying-Hsin Lu[6], Ting-Chang Chang[6], Li-Wei Tu[6], Hsisheng Teng[7], Yi-Chun Chen[2], Chia-Hao Chen[3], and Chung-Lin Wu[2*]

[1]*Center for Micro/Nano Science and Technology, National Cheng Kung University, Tainan 70101, Taiwan*

[2]*Department of Physics, National Cheng Kung University, Tainan 70101, Taiwan*

[3]*National Synchrotron Radiation Research Center, Hsinchu 30076, Taiwan*

[4]*National Nano Devices Laboratories, National Applied Research Laboratories, Tainan 741, Taiwan*

[5]*Department of Physics, National Tsing-Hua University, Hsinchu 30013, Taiwan.*

[6]*Department of Physics, National Sun Yat-Sen University, Kaohsiung 804, Taiwan*

[7]*Department of Chemical Engineering, National Cheng Kung University, Tainan 70101, Taiwan*

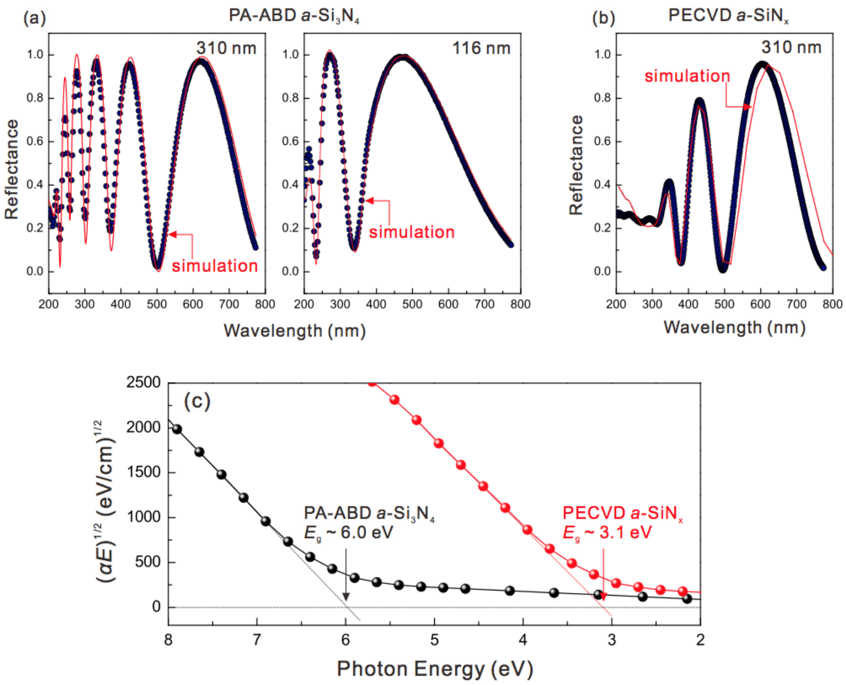

Figure. S1 (color). Optical *E*g characterization from reflectance spectra: Optical band gap measurement of PA-ABD *a*-Si_3N_4 and PECVD *a*-SiN_x obtained from reflection measurement by extracting extinction coefficient *k* from the normal-incidence reflection measurement to calculate the absorption coefficient $\alpha = 4\pi k/\lambda$. The reflectance data for PA-ABD *a*-Si_3N_4 and PECVD *a*-SiN_x shown in (**a**) and (**b**) were obtained from silicon nitride layer on polished Si substrates. The simulated reflectance (red curves in (**a**) and (**b**)) is obtained by applying the Fresnel equation.[1] The normal-incidence reflectivity can be written as $\mathrm{R} = |r|^2 = [(r_1 + r_2 e^{i\varphi})/(1 + r_1 r_2 e^{i\varphi})]^2$, where $r_1 = (n_{air} - \tilde{n}_{SiNx})/(n_{air} + \tilde{n}_{SiNx})$ and $r_2 = (\tilde{n}_{SiNx} - \tilde{n}_{Si})/(\tilde{n}_{SiNx} + \tilde{n}_{Si})$ (n_{air}, $\tilde{n}_{SiNx}$, and $\tilde{n}_{Si}$ are reflective index for air, SiNx, and Si,[2] respectively) the amplitude reflection coefficient of interfaces, and $\varphi = 2\pi \tilde{n}_{SiNx} d/\lambda$ (d is the thickness of SiNx is the phase difference due to changes in the optical path.

(**c**) The optical band gap were determined by plotting the absorption coefficient (α) against the photon energy (eV) and taking the intercept of the extrapolation to zero absorption with photon energy axis (dashed lines[3] for PA-ABD *a*-Si_3N_4 and PECVD *a*-SiN_x.

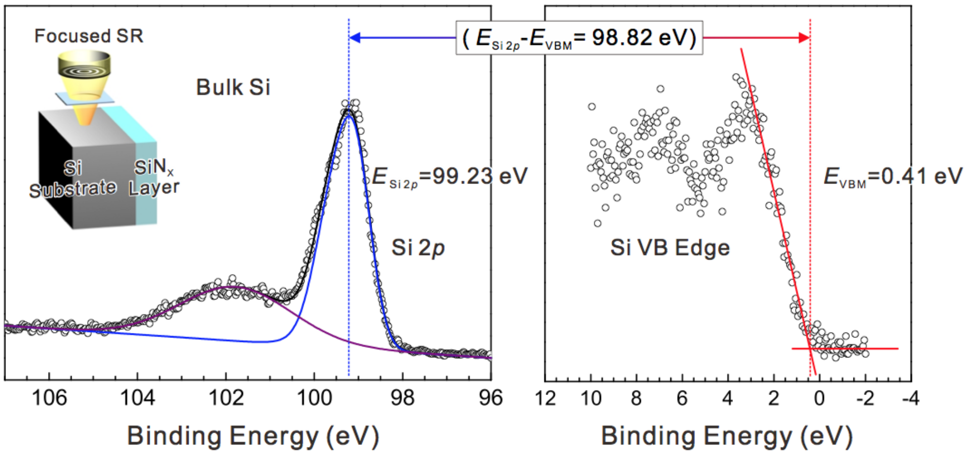

Figure. S2 (color).

Figure. S2 (color). *μ*-PES characterization on cleaved Si substrates: *μ*-PES spectra of the Si 2*p* core-level and valence band taken on the Si bulk region of the cross-sectional PA-ABD *a*-Si_3N_4/Si(111) sample. By linearly extrapolating the leading edge of the valence-band spectrum to the base line, the VBM position can be precisely located at 0.41 eV. These two spectra reveal the Si bulk energy separation of 98.82 eV between the VBM and the Si 2*p* core level.

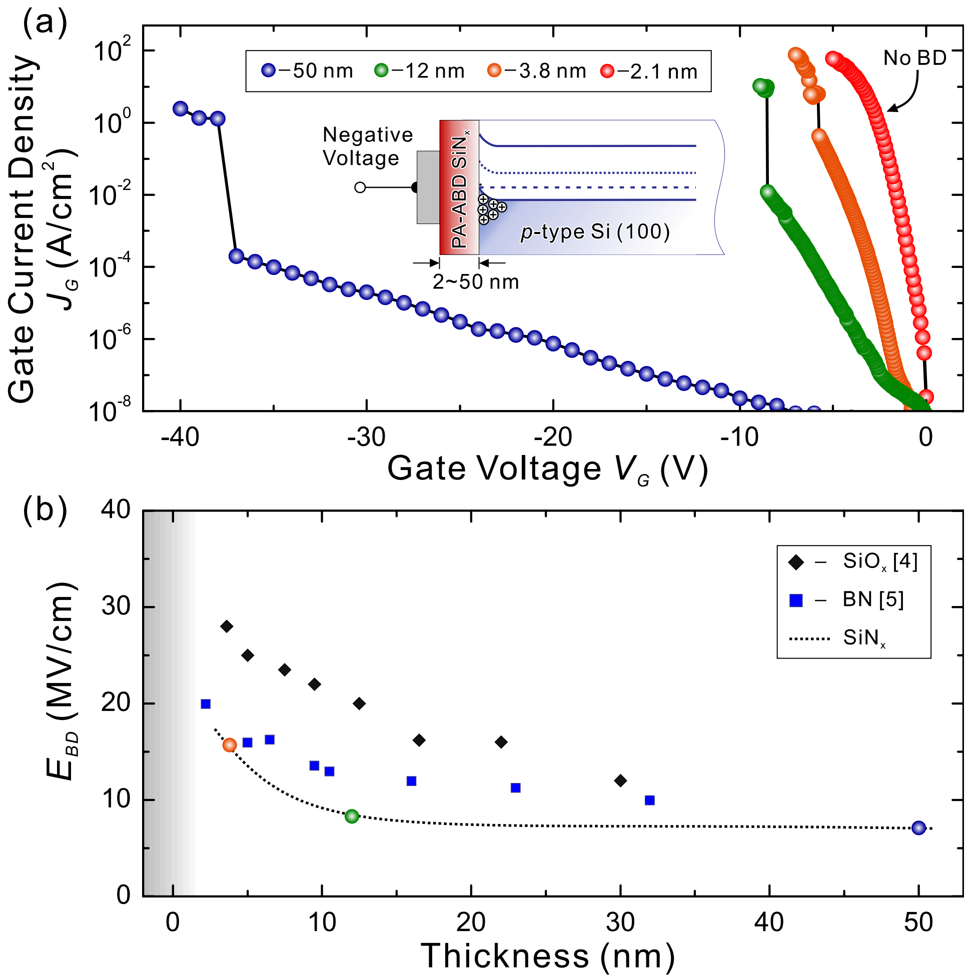

Figure. S3 (color). Leakage current and breakdown fields characterizations: (**a**) Leakage gate current densities and breakdown fields of PA-ABD *a*-Si_3N_4/(100) with various thickness. (**b**) Breakdown field as a function of dielectric thickness for the corresponding a-SiNx shown in (**a**). Previously reported data for SiO_2[4] and single crystalline BN[5] are plotted to show that the increases in E_{BD} for thinner dielectric thickness are also observed for amorphous and crystalline materials. The behavior is resulted from the tunneling currents flowing though the dielectric due to Fowler-Nordheim tunneling or direct tunneling, and thus lower the strength of local electric field in dielectric layer.

References

[1] Anders, H. (ed.) *Thin Films in Optics* (Focal Press, 1967).

[2] Aspnes D. E.; Studna, A. A. Dielectric functions and optical parameters of Si, Ge, GaP, GaAs, GaSb, InP, InAs, and InSb from 1.5 to 6.0 eV. *Phys. Rev. B* **27**, 985 (1983).

[3] Tauc, J. (ed.) *Amorphous and Liquid Semiconductors* (Plenum, 1974).

[4] Harari, E. Conduction and trapping of electrons in highly stressed ultrathin films of thermal SiO_2. *Appl. Phys. Lett.* **30**, 601 (1977).

[5] Hattori, Y.; Taniguchi, T.; Watanabe, K.; Nagashio, K. Layer-by-layer dielectric breakdown of hexagonal boron nitride. *ACS Nano* **9** (1), 916-921 (2015).
